# Review and Analysis of Heat Transfer in Spacer-Filled Channels of Membrane Distillation Systems

**DOI:** 10.3390/membranes13100842

**Published:** 2023-10-22

**Authors:** Sebastian Schilling, Heike Glade

**Affiliations:** Engineering Thermodynamics, University of Bremen, 28359 Bremen, Germany

**Keywords:** membrane distillation, modelling, heat transfer, spacer-filled channel, brine concentration

## Abstract

Membrane distillation (MD) is an attractive process for the concentration of seawater brines. Modelling and simulation of membrane distillation processes requires a better knowledge of the heat transfer coefficients in spacer-filled channels which are usually determined by applying empirical correlations for the Nusselt number. In this study, first, a comprehensive literature review on heat transfer correlations was conducted. It was found that the empirical correlations often used for MD simulation result in strongly varying Nusselt numbers that differ by up to an order of magnitude at low Reynolds numbers. Then, heat transfer in spacer-filled channels was investigated experimentally in a membrane distillation system using an aluminum plate instead of a flat-sheet membrane. Numerous tests were carried out with sodium chloride solutions in a wide range of salinities, between 1 g/kg and 95 g/kg, and temperatures, between 30 °C and 80 °C, yielding high heat transfer coefficients in a range of 1500 to 8300 W/(m^2^K) at relatively low Reynolds numbers, between 100 and 1500, clearly showing the influence of the spacers on heat transfer. A new empirical Nusselt correlation ($Nu=0.158{Re}^{0.652}{Pr}^{0.277}$) was derived which represents the experimental data with a deviation of 10% and is valid for 100<Re<1500 and 2<Pr<7. Computational fluid dynamics simulations were performed to analyze the variations of the fluid properties across the boundary layer due to temperature differences. The simulations showed only minor deviations of the heat transfer coefficients in the hot and cold fluid channels for small driving temperature differences.

## 1. Introduction

Membrane distillation (MD) has attracted considerable attention as a potential alternative to conventional thermal and membrane-based desalination technologies in various fields of application, such as brine concentration, which is needed for water treatment strategies like zero liquid discharge and minimal liquid discharge [1]. Membrane distillation is a non-isothermal process that separates vaporizable liquids from non-volatiles such as colloids, proteins, mineral ions and other dissolved solids. The process is driven by a temperature gradient across a microporous hydrophobic membrane, establishing a vapor pressure difference between the feed solution and the permeate [2,3]. The feed solution is always in direct contact with the membrane surface. Volatile compounds of the feed solution evaporate and the vapor molecules are forced through the membrane pores from the feed to the permeate side. Due to the hydrophobicity of the membrane material and the surface tension of water, the membrane pores are not wetted, and evaporation takes place at their openings.

Different approaches for establishing the driving force across the membrane and other process requirements have led to the development of various permeate channel configurations [2,3,4,5]. Depending on the process configuration, the vapor at the permeate side of the membrane can be condensed in different ways. The vapor directly condenses into a liquid (direct contact membrane distillation) or it diffuses through an air gap and condenses on a cooled plate (air gap membrane distillation). In order to condense the permeate vapor externally, it can be swept out of the module by a gas stream (sweeping gas membrane distillation), or it is forced out of the module by an applied vacuum (vacuum membrane distillation) at a pressure that is lower than the saturation pressure of volatile molecules to be separated from the feed solution [2]. Membrane distillation can be performed in plate-and-frame modules, in tubular modules, or in hollow fiber modules. While spacers in the feed and permeate channel of plate-and-frame modules ensure a constant flow cross-section, spacers are not used in tubular modules and the flow is unrestricted.

Membrane distillation offers attractive benefits compared to conventional desalination processes. Relying on a thermal principle, MD is capable of concentrating feed solutions up to their saturation point without any significant permeate flux decline. The hydrophobic membrane allows only the passage of vapors and retains all non-volatile streams on the retentate side. Thus, the permeate is theoretically 100% pure of solid or non-volatile contaminants. Moreover, MD operates at low temperatures and can be driven by solar thermal energy, geothermal energy or waste heat associated with low-temperature industrial streams [4].

Seawater desalination for the production of fresh water has been the most common application considered in MD research and pilot testing [6,7,8]. In addition, MD has been used in brine concentration and waste water treatment (e.g., recovery of hypersaline waste water from hydraulic fracturing) as well as in the chemical and food industries [9]. Membrane distillation has been identified as a promising process for small-scale, self-sufficient and environmentally friendly decentralized desalination systems using low-grade waste heat or renewable energy supplies [10]. The growing need for brine concentration and zero liquid discharge systems opens up significant opportunities for membrane distillation in waste water treatment. Since reverse osmosis can be applied only to feed waters within a limited salinity range, membrane distillation has recently emerged as an alternative zero liquid discharge technology to further concentrate waste waters after the reverse osmosis stage [4,11].

Although membrane distillation has not been widely implemented in the desalination industry, research interest is increasingly growing. Most of the available literature (e.g., [2,6]) deals with theoretical models, especially on topics related to heat transfer and mass transfer [3]. Membrane distillation is a separation process governed by coupled heat and mass transfer, i.e., mass transfer through the pores of the membrane and heat transfer through both the membrane matrix and its pores are involved simultaneously [3]. Fluid boundary layers adjoining the feed and permeate membrane sides are formed due to convective transport mechanisms under cross-flow conditions. When simulating heat and mass transfer in MD, a resistance-in-series method is often used [12,13]. This approach incorporates various resistances, starting with the feed-side boundary layer resistance, which can be estimated using appropriate convective transfer coefficients. Subsequently, there is a resistance associated with conduction through the membrane material, along with the simultaneous diffusion of vapor described by the dusty-gas model. The resistances at the permeate side differ according to the channel configuration [5,12,13]. 

In order to determine the heat transfer coefficient in the fluid boundary layers adjacent to the membrane surface, a large variety of empirical correlations for the Nusselt number have been reported in the literature [5]. However, it is crucial to select an appropriate correlation that describes the heat transfer mechanism adequately [13]. The majority of the empirical correlations have been derived for plain, smooth and rigid metal heat exchanger surfaces, although hydrodynamics in plate-and-frame modules may also be influenced by spacers that destabilize the flow and create eddy currents, even in the laminar regime, enhancing momentum and heat and mass transfer [5,14,15]. Thus, spacer-filled channels can have significantly different heat transfer characteristics, because fluid velocities and critical Reynolds numbers are substantially lower compared to those in conventional heat exchangers or empty channel applications. There are only a few studies [16,17,18,19,20,21] that present an empirical correlation for the Nusselt number derived from measurements in MD systems. Furthermore, the Prandtl number is usually kept unchanged during the fitting [16,17,18,20,21], and therefore may not adequately describe the changes in fluid properties with temperature.

For simulating membrane distillation, a better knowledge of heat transfer is also of the utmost importance because mass transfer is often calculated analogously. In this study, a comprehensive literature review was carried out on heat transfer in spacer-filled channels. Then an experimental analysis of heat transfer in a plate-and-frame module with spacer-filled channels was performed. Based on measurements of the overall heat transfer coefficient for a variety of flow conditions, temperatures and fluid properties, a multi-dimensional regression was performed to derive a correlation for the Nusselt number that describes heat transfer mechanisms in a plate-and-frame module with spacer-filled fluid channels for the concentration of seawater brines.

## 2. Theoretical

Membrane distillation is a process of combined heat transfer and mass transfer. The driving force for mass transfer is described by the difference of the vapor pressures $\Delta p_{i}$ across the hydrophobic membrane that is established by a temperature difference Δϑ. The transmembrane mass flux ${\dot{m}}_{i}$ of component i is given by

(1) $${\dot{m}}_{i}=C_{m}\left[p_{i}\left(\vartheta_{fm},c_{fm}\right)-p_{i}\left(\vartheta_{pm},c_{pm}\right)\right]$$

where $C_{m}$ is a membrane characteristics parameter which also depends on the operating parameters and the actual mass transfer mechanisms and can be evaluated according to the dusty-gas model. The vapor pressure difference is a function of the temperatures and concentrations at the feed-side membrane surface $\vartheta_{fm},c_{fm}$ and the permeate-side membrane surface $\vartheta_{pm},c_{pm}$, respectively. In fact, the temperature at the membrane surface is lower than in the bulk of the feed due to heat transfer through the membrane matrix and the pores. In contrast, non-volatile components of the feed are not evaporated and therefore accumulate on the membrane surface. These phenomena are known as temperature polarization and concentration polarization. Furthermore, diffusive fluxes, as given by Equation (1), can be superposed by viscous fluxes based on the absolute pressure difference in various process configurations [2,3,4,5,12,16].

The well-known Clausius–Clapeyron [2,3] or Antoine [22] equations are used to determine the temperature dependence of the vapor pressure [13]. In addition to the temperature, the interfacial vapor pressures are also a function of the fluid composition and concentrations $c_{im}$ at the membrane surface. To account for the non-ideality of a liquid mixture, the activity coefficients of the components in the solution can be considered [5,12]. In particular for concentrated seawater, the literature [23,24,25] contains empirical correlations for the vapor pressure (and other thermophysical properties). 

The transfer of heat in an MD module is divided into the three following steps: (i) heat transfer through the feed boundary layer, (ii) heat transport through the membrane and (iii) heat transport through the permeate boundary layer [2]; these will be examined in detail in the following.

### 2.1. Transport across the Membrane

The heat transfer through the membrane is composed of conduction through the membrane matrix material and the latent heat transported by vapor diffusion [4,5,12]. Hence, the heat flux ${\dot{q}}_{m}$ through the membrane is given by

(2) $${\dot{q}}_{m}={\dot{m}}_{i}\Delta {\bar{h}}_{vap}+\frac{k_{m}}{\delta_{m}}\left(\vartheta_{fm}-\vartheta_{pm}\right)$$

where $\Delta {\bar{h}}_{vap}$ is the specific enthalpy of vaporization, $\delta_{m}$ is the membrane thickness and $k_{m}$ is the thermal conductivity of the membrane.

For determining the effective thermal conductivity of the porous membrane $k_{m}$, a combined thermal conductivity of the solid $k_{m,s}$ and gaseous $k_{m,g}$ phases is considered. The thermal conductivity of a bulk polymer depends on its crystallinity, spatial arrangement and stretching, as well as the temperature level. It can vary between 0.1 and 0.5 W/(m K) [26]. The thermal conductivity of the gaseous phase, which is composed of vapor and air trapped inside the pores, can be estimated. However, the composition of the gas phase has a negligible influence, as the thermal conductivities of vapor and air are similar [16,27].

In order to calculate the effective thermal conductivity of a material composed of two (or more) components, various models can be found in the literature [28]. Many authors [2,3,12,13,22,29] apply an iso-strain model. Considering the thermal resistance of the solid and the gaseous phase as parallel to the heat flow, the effective thermal conductivity is calculated by

(3) $$k_{m,iso-strain}=\sum_{i=1}^{n}\varepsilon_{i}\cdot k_{i}=\varepsilon_{m}k_{m,g}+\left(1-\varepsilon_{m}\right)k_{m,s}$$

where $\varepsilon_{m}$ is the porosity of the membrane. According to Krischer et al. [30], Equation (3) represents an extreme case in which the solid is considered as a continuous network. Phattaranawik et al. [27] pointed out that the application of the iso-strain model gives too high effective thermal conductivities. Therefore, the iso-stress model was suggested [2,3,16,27,31], in which the thermal resistances of the membrane and the vapor/air-mixture are arranged in series

(4) $$k_{m,iso-stress}={\left(\sum_{i=1}^{n}\frac{\varepsilon_{i}}{k_{i}}\right)}^{-1}={\left(\frac{\varepsilon_{m}}{k_{m,g}}+\frac{1-\varepsilon_{m}}{k_{m,s}}\right)}^{-1}.$$

In this resistance-in-series approach, the fluid is considered as a continuous phase comparable to powdery, granular or fibrous materials [30].

In fact, the iso-strain and iso-stress models represent two extreme cases of thermal resistances considered as purely parallel or in-series, respectively. Krischer et al. [30] suggested a superposed iso-strain/iso-stress approach given by

(5) $$k_{m,iso-superposed}={\left(\frac{1-C}{k_{m,iso-strain}}+\frac{C}{k_{m,iso-stress}}\right)}^{-1},$$

where C is a constant with 0<C<1 that assumes an effective thermal conductivity as weighted average of the maximum ($k_{m,iso-strain}$) and minimum ($k_{m,iso-stress}$) thermal conductivities [28,31,32]. For homogeneous membrane structures, Nikolaus [32] suggested a value of C=0.5.

Additionally, it was proposed to calculate the effective thermal conductivity by a geometric mean approach [28] expressed as

(6) $$k_{m,geometric\;mean}=\prod_{i=1}^{n}k_{i}^{\varepsilon_{i}}=k_{m,g}^{\varepsilon_{m}}\cdot k_{m,s}^{\left(1-\varepsilon_{m}\right)}.$$

Another proposed model for calculating the effective thermal conductivity is the flux-law model [27], which represents an application of the Maxwell solution of the Laplace equation for the temperature field [31]. The effective thermal conductivity can be calculated as

(7) $$k_{m,flux\;law}=k_{m,g}\left[\frac{1+2\cdot \left(1-\varepsilon_{m}\right)\frac{k_{m,s}-k_{m,g}}{k_{m,s}+2k_{m,g}}}{1-\left(1-\varepsilon_{m}\right)\frac{k_{m,s}-k_{m,g}}{k_{m,s}+2k_{m,g}}}\right]\;.$$

**Figure 1:**
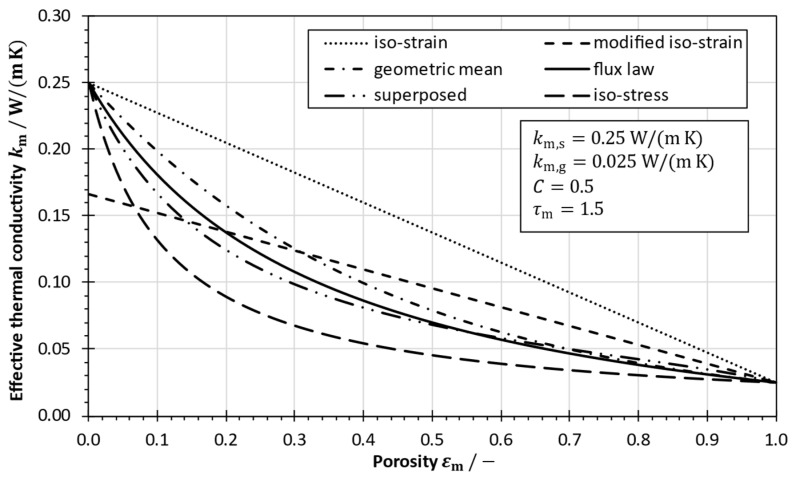
Effective thermal conductivity of a membrane distillation (MD) membrane as a function of the porosity, based on model equations for composed materials.

A further approach is based on the assumption that the effective path length for conductive heat transport in the solid phase is longer than the actual membrane thickness. Hence, the incorporation of the membrane’s tortuosity results in a modified iso-strain model [16,33],

(8) $$k_{m,modified\;iso-strain}=\varepsilon_{m}k_{m,g}+\left(1-\varepsilon_{m}\right)\frac{k_{m,s}}{\tau_{m}}$$

with the membrane’s tortuosity being $\tau_{m}\geq 1$ [16,33]. A comparison of the different proposed model equations for composed materials is given in Figure 1.

As pointed out by Phattaranawik et al. [27], a comparison of measurement data from Izquierdo-Gil et al. [34] with calculated effective thermal conductivities revealed that the iso-strain model leads to an overestimation, and the iso-stress model provides the best agreement. In contrast, Qiu et al. [35] reported that their measurements showed good agreement with the iso-strain model. Furthermore, a comprehensive analysis of the effective thermal conductivities of MD membranes was performed by García-Payo and Izquierdo-Gil [28], who compared various models with measurements of different membrane samples. They concluded that the traditionally used approach according to the iso-strain (resistances in parallel) model leads to a significant overestimation of the effective thermal conductivity and the iso-stress model (resistances in series) results in an underestimated effective thermal conductivity. The other proposed models result in effective thermal conductivities between these extreme cases, with the flux-law model (Maxwell solution of Laplace equation) yielding the best agreement with the measured data for highly porous membranes ($\varepsilon_{m}>0.6$). In consequence, the flux-law model was suggested for preferred use, as it better reflects the physical reality and it does not include additional (unknown) parameters [28]. Recently, machine-learning techniques were used to estimate effective thermal conductivity, which may improve the precision of future models [36].

### 2.2. Transport across the Fluid Boundary Layers

Heat flux ${\dot{q}}_{i}$ in the fluid boundary layers is usually described by forced convection,

(9) $${\dot{q}}_{f}=h_{f}\;\left(\vartheta_{f}-\vartheta_{fm}\right)\;\mathrm{and}\;{\dot{q}}_{p}=h_{p}\;\left(\vartheta_{pm}-\vartheta_{p}\right)$$

where the temperature differences are calculated between the fluid bulk $\vartheta_{f}$ and $\vartheta_{p}$ and the fluid at the membrane surface $\vartheta_{fm}$ and $\vartheta_{pm}$ for feed and permeate, respectively. The heat transfer coefficients $h_{f}$ and $h_{p}$ in the feed and permeate boundary layers, respectively, can be determined using appropriate empirical correlations for the corresponding Nusselt numbers

(10) $$Nu_{f}=\frac{h_{f}d_{h}}{k_{f}}\;\mathrm{and}\;Nu_{p}=\frac{h_{p}d_{h}}{k_{p}}$$

where $d_{h}$ is the hydraulic diameter and $k_{f}$ and $k_{p}$ are the liquid thermal conductivities of feed and permeate, respectively. For non-circular cross-sections the hydraulic diameter is determined according to

(11) $$d_{h}=4\frac{A}{P}$$

where A is the flow cross-sectional area and P is the wetted perimeter of the channel [37]. If a net-type spacer is used in the channel, the hydraulic diameter is given by

(12) $$d_{h}=\frac{4\varepsilon_{S}}{\frac{2}{\delta_{S}}+\left(1-\varepsilon_{S}\right)\frac{4}{d_{S}}}$$

where $\varepsilon_{S}$ is the voidage of the spacer net, $\delta_{S}$ is its thickness and $d_{S}$ denotes the diameter of a spacer filament [16,38,39,40].

Correlations for the Nusselt number for forced convection usually have the form

(13) $$Nu_{forced}=C_{1}Re^{C_{2}}{Pr}^{C_{3}}{\left(d_{h}/L\right)}^{C_{4}}$$

involving empirical constants $C_{i}$ as well as the Reynolds number Re,

(14) $$Re=\frac{\bar{u}d_{h}}{\nu }$$

and the Prandtl number Pr,

(15) $$Pr=\frac{\nu \rho c_{p}}{k}$$

where $\bar{u}$ denotes the mean fluid velocity, ν is the kinematic viscosity, ρ is the density and $c_{p}$ is the specific isobaric heat capacity of the fluid. The thermophysical properties are to be evaluated at a mean temperature [37]. The ratio of the hydraulic diameter to the length $d_{h}/L$ characterizes the geometry of the fluid channel and can affect the pressure drop. Normally, L denotes the (entire) length of the flow channel. However, in the case of spacer-filled channels it seems more reasonable to consider the mesh size $l_{S}$, the mean spacing between two individual adjacent filaments, for the calculation of the channel geometry [40,41,42].

The Reynolds number allows the division of the flow regime into laminar and turbulent flows. In circular tubes, flow is laminar for Re≤2300 and fully turbulent for $Re\geq 10^{4}$. Between these critical values, flow is considered to be in transition [43]. For spacer-filled channels, the critical Reynolds number is significantly lower due to the formation of recirculating vortices by the spacer filament structures. These instabilities in the flow regime were found at Reynolds numbers of about Re>180 [44,45,46], questioning the application of conventional empirical correlations for the Nusselt number. In fact, Mojab et al. [47] pointed out that flow in spacer-filled channels becomes oscillatory at Re∼250 and is laminar-unsteady-periodic until Re<300, while it becomes fully unsteady for Re>300. Similar observations were reported by Qamar et al. [48] who found laminar flow below Re<250. Increasing the Reynolds number to Re>250 leads to unsteadiness due to vortex formation and to a fully turbulent flow for Re>350. Moreover, it was shown that onset of vortex formation and turbulent flow also depend on the surface roughness and on mesh size [49,50].

In the presence of natural convection in a forced convective flow, the heat transfer rate is enhanced [13]. The general form of a correlation for the Nusselt number for natural convection is given by

(16) $$Nu_{free}=C_{5}{Gr}^{C_{6}}{Pr}^{C_{7}}$$

where Gr is the Grashof number expressed as

(17) $$Gr=\frac{g\beta_{V}\left(\vartheta_{W}-\vartheta \right)H^{3}}{\nu^{2}}\;.$$

Here, g denotes gravitational acceleration, $\beta_{V}$ is the isobaric volume expansion coefficient and H the height, respectively. The temperature difference $\left(\vartheta_{W}-\vartheta \right)$ refers to the difference between the bulk stream and the membrane surface in MD. The thermophysical properties are to be evaluated at a mean temperature $\bar{\vartheta }=0.5\;\left(\vartheta +\vartheta_{W}\right)$ [51]. The Grashof number Gr characterizes the fluid dynamics arising through buoyancy forces caused by a temperature gradient. Natural convection is often described in terms of the Rayleigh number Ra=Gr·Pr [51]. A decisive quantity for mixed convection is the Richardson number $Ri=Gr/Re^{2}$, which is in the order of 1 for equal buoyancy and inertial forces [52]. Therefore, forced convection is assumed to dominate the heat transfer for Ri≪1, while Ri≫1 indicates a predominant natural convection [53]. Depending on the direction of the pressure-driven flow relative to the buoyancy forces, the resulting heat transfer may be mutually stimulated or dampened [53,54]. Hence, a combined Nusselt number can be calculated [53,54,55,56] as

(18) $$Nu=\sqrt[{C}]{\left|Nu_{forced}^{C}\left(Re,Pr\right)\pm Nu_{free}^{C}\left(Gr,Pr\right)\right|}$$

where the exponent C is generally C=3 [54]. However, C=4 was used for longitudinal flow over horizontal plates, cylinders or spheres [53]. For free and forced flow heading in the same direction, the positive sign is valid. However, heat transfer can be reduced—as indicated by a negative sign—if counter-current flow is present [53,54]. Empirical correlations for mixed forced and natural convection in MD were given by Gryta et al. [13]. 

In addition to the flow regime and the fluid properties, the geometry of the fluid channel determines the empirical coefficients in Nusselt correlations. Various correlation equations have been used in MD modelling (cf. Table A1). In the following, an overview of frequently used empirical equations for the Nusselt number is given, whereby the basic forms without modification (see Equations (39)–(43)) are summarized. It is important to note that these correlations were not originally derived for an application in MD but for rigid metal surfaces in heat exchangers, e.g., for the flow through pipes or the flow over plates. Each empirical equation in its original application is derived for a specific range of Reynolds and Prandtl numbers (and $d_{h}/L$) that can be found in the corresponding literature.

In membrane distillation modelling, most often the well-known Lévêque [57] equation

(19) $$\bar{Nu}=\frac{\int_{0}^{L}Nu_{x}\;dx}{\int_{0}^{L}dx}=1.62{\left(Re\;Pr\;d_{h}/L\right)}^{0.333}$$

or the Sieder-Tate [58] correlation

(20) $$\bar{Nu}=1.86{\left(Re\;Pr\;d_{h}/L\right)}^{0.333}$$

has been used to calculate the mean Nusselt number for laminar flow on the lumen side in tubular membranes [18,58,59,60,61,62]. Although the factor $C_{1}=1.62$ in the Lévêque equation results from the Hagen–Poiseuille law for laminar flow in circular tubes (ζ=64/Re) [63], both equations have also been widely used for rectangular ducts in the flat-sheet MD configuration [13,22,27,39,64,65,66,67,68,69,70,71,72,73,74]. Furthermore, heat transfer coefficients for laminar flow over a flat plate can be described with an equation given by Gröber et al. [75]. The mean Nusselt number is given by [75,76]

(21) $$\bar{Nu}=0.664Re^{0.5}Pr^{0.333}.$$

The Gröber equation was suggested for the calculation of heat transfer coefficients in fluid channels with spacers that do not induce a change in flow direction [39]. Considering pipe flow between parallel flat plates, Pohlhausen [77] derived a correlation for the mean Nusselt number by integration along the flow path length L, resulting in

(22) $$\bar{Nu}=0.664Re^{0.5}Pr^{0.333}{\left(d_{h}/L\right)}^{0.5}.$$

Da Costa et al. [40,78] developed an empirical equation for spacer-filled channels by modifying the Gröber equation [75] as

(23a) $$\bar{Nu}=0.644f_{S}{Re}^{0.5}{Pr}^{0.333}{\left(2d_{h}/l_{S}\right)}^{0.5}$$

with the mesh size $l_{S}$ and a factor $f_{S}$ accounting for the spacer properties such as voidage $\varepsilon_{S}^{}$, thickness $\delta_{S}$, mesh angle Φ, and filament diameter $d_{S}$:

(23b) $$f_{S}=1.654{\left(d_{S}/\delta_{S}\right)}^{-0.039}\varepsilon_{S}^{0.75}{\left(\sin\left(\Phi /2\right)\right)}^{0.086}.$$

Another Nusselt number correlation for fully developed laminar flow was given by Hausen [79]:

(24) $$\bar{Nu}=Nu_{i}+\frac{0.0668{Re\;Pr\;d}_{h}/L}{1+0.04{\left({Re\;Pr\;d}_{h}/L\right)}^{0.667}}$$

where $Nu_{i}$ is a constant depending on the boundary conditions [12,18]. For constant wall temperature $\left(\vartheta_{W}=\mathrm{const}\right)$ or constant heat flux $\left({\dot{q}}_{W}=\mathrm{const}\right)$, $Nu_{i}$ is $Nu_{\vartheta_{W}=\mathrm{const}}=3.66$ and $Nu_{{\dot{q}}_{W}=\mathrm{const}}=4.36$, respectively. It is important to note that these boundary constraints are valid only for circular tubes and might be modified for rectangular fluid channels [80]. With increasing fluid velocity, the occurrence of flow instabilities promotes turbulences, reducing the boundary layer thickness. As a result, the heat transfer coefficient is enhanced. Thus, Mengual et al. [18] described the heat transfer in the transition regime as

(25) $$\bar{Nu}=0.116\left(Re^{0.667}-125\right){Pr}^{0.333}.$$

For the calculation of heat transfer coefficients in fully developed turbulent flow, the Colburn [81] equation

(26) $$\bar{Nu}=0.023Re^{0.8}{Pr}^{0.333}$$

has been widely applied [19,27,60,82,83,84]. Furthermore, the modification according to Dittus and Boelter [85], which takes into account an exponent C to the Prandtl number depending on the direction of the heat flux,

(27) $$\bar{Nu}=0.023Re^{0.8}{Pr}^{C}\;\mathrm{with}\;C=\left\{\begin{array}{c} 0.4\;:\;\vartheta_{W}>\vartheta \;(heating)\;\\ 0.3\;:\;\vartheta_{W}<\vartheta \;(cooling) \end{array}\right.$$

has been used to predict the heat transfer coefficient in turbulent flow in tubular [18,61,86] and flat-sheet [65,73,74,86] configurations. Alternatively, the Sieder–Tate [58] correlation for turbulent flow [18,22,27,71]

(28) $$\bar{Nu}=0.027Re^{0.8}{Pr}^{0.333}$$

or its modification with an entrance length $Nu/Nu_{\infty }=4/3\cdot {\left(d_{h}/L\right)}^{0.055}$ [27,87] yielding

(29) $$\bar{Nu}=0.036Re^{0.8}{Pr}^{0.333}{\left(d_{h}/L\right)}^{0.055}$$

has been applied in turbulent flow in circular and rectangular configurations. Moreover, Incropera et al. [88] developed a correlation

(30) $$\bar{Nu}=0.13Re^{0.64}{Pr}^{0.38}$$

for turbulent flow through rectangular channels for Reynolds numbers 5000<Re<14,000. Winter [16] investigated heat transfer in spacer-filled channels by comparing two different spacer types with the empty channel. In his study, the highest heat transfer was achieved with the thinner (2 mm) spacer, yielding

(31) $$\bar{Nu}=0.106Re^{0.695}{Pr}^{0.333}.$$

Another equation that is applied to MD was originally derived by Gnielinski [89] from analogy of momentum transport in fully developed turbulent flow through circular tubes [22,27,90]:

(32) $$\bar{Nu}=\frac{\left(\zeta /8\right)\left(Re-1000\right)Pr}{1+12.7\sqrt{\zeta /8}\left({Pr}^{0.667}-1\right)}\;.$$

The friction factor ζ indicates the intensity of the pressure drop inside tubes and is subjected also to the roughness of the tube wall [91]. It can be determined using the equations shown in Table 1 when assuming technically smooth surfaces. Although the Konakov equation (cf. Equation (36)) was recommended for industrial use [37], the Filonienko equation (cf. Equation (37)) has a broader range of applicability and was applied successfully in MD simulations [27,90]. However, compared to the correlations mentioned beforehand, the Taler equation (cf. Equation (38)) shows a higher accuracy, especially at increased Reynolds numbers ($Re>2\cdot 10^{4}$). In addition, it provides a large range of applicability and thus may be preferred [92]. It is important to note that the friction factors shown here are valid for empty (smooth) tubular channels and differ from friction factors in spacer-filled channels that must be determined experimentally [16,40].

**Figure 2:**
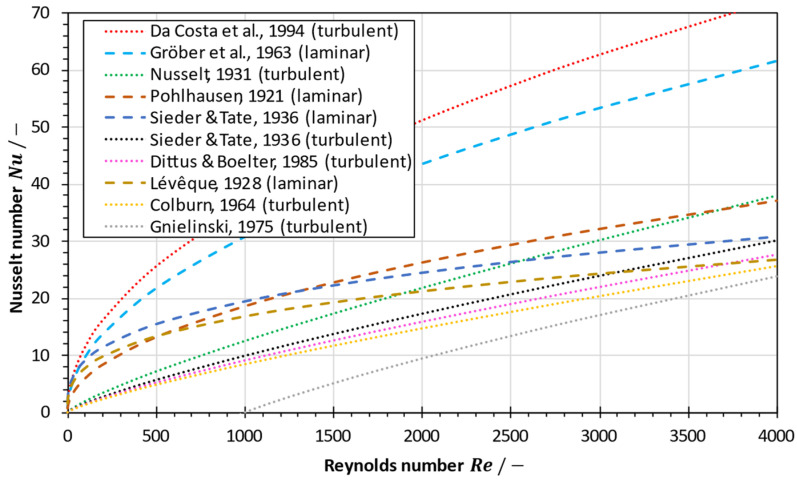
Comparison of Nusselt correlations used in MD modelling for a flat-sheet configuration with spacer-filled channels. Calculated for seawater $(S=35\;g/kg,\;\vartheta_{f}=60\;{}^{\circ}C,\;Pr=3.15,$ $d_{h}/l_{S}=0.365$) [40,57,58,75,77,81,85,87,89].

Figure 2 shows a comparison of the Nusselt number calculated with the correlations given in Equations (19)–(23), (26)–(29) and (32) for seawater at 60 °C (Pr=3.15) in a flat-sheet module with spacer-filled channels. The empirical correlations often used for MD modelling and simulation result in strongly varying Nusselt numbers that differ by up to an order of magnitude at low Reynolds numbers. As depicted in Figure 2, the Gröber equation (cf. Equation (21)) and its modification by Da Costa et al. [40,78] (cf. Equation (23)) lead to significantly higher Nusselt numbers than those obtained by the classical Lévêque (cf. Equation (19)), Sieder-Tate (cf. Equation (20)) or Pohlhausen equations (cf. Equation (22)) for laminar flow when used for a flat-sheet configuration with spacer-filled channels. Therefore, great caution must be exercised when applying these equations under boundary conditions for which they were not established. Furthermore, due to the strong dependence of the flow on the complex channel geometry in membrane distillation modules, any particular Nusselt equation must be carefully validated for a given scenario [16]. 

To improve the quality of calculations of the heat transfer coefficients for a specific channel geometry, many authors [14,16,17,18,40,95] use a basic form of the Nusselt number correlation and identify the respective constants by fitting them to their own experimental data. A review of empirical equations for the Nusselt number used in membrane distillation modelling is given in Table A1 in Appendix A.

When applying the empirical Nusselt correlations to practical heat transfer problems, it is a common practice to consider the effects of temperature differences or the evolution of hydraulic and thermal profiles in an entrance length of the fluid channel. When high temperature differences between the fluid bulk and the heat transfer wall are present, a correction factor for the change of fluid properties must be considered. Conventionally, the correction factor takes the dynamic viscosities into account [58,96]:

(39) $$\frac{\bar{Nu}}{{\bar{Nu}}_{0}}={\left(\frac{\eta }{\eta_{W}}\right)}^{0.14}$$

where ${\bar{Nu}}_{0}$ is the Nusselt number determined at the liquid bulk mean temperature and $\eta /\eta_{W}$ is the ratio of dynamic viscosities of the liquid in the bulk and near the heat transfer wall. Recent practice prefers the adaption of the Prandtl number ratio instead of the viscosity ratio for the liquid at the corresponding temperatures. Hufschmidt and Burck [97] found a factor of ${\left(Pr/{Pr}_{W}\right)}^{0.11}$ for turbulent flow. Since the values measured by Sieder and Tate [58] are scattered, it was proposed to extend ${\left(Pr/{Pr}_{W}\right)}^{0.11}$ also to laminar flow [37]. Hence, the correction equation is given by

(40) $$\frac{\bar{Nu}}{{\bar{Nu}}_{0}}={\left(\frac{Pr}{{Pr}_{W}}\right)}^{0.11}\;.$$

It can be used in laminar and turbulent flow, and it is valid for $0.1<Pr/{Pr}_{W}<10$ [37,97].

In an entrance region at the beginning of the channel, the temperature gradients are still forming. Thus, the temperature profile is developing, which enhances the heat transfer. This enhancement can be described in terms of the ratio of hydraulic diameter $d_{h}$ to the length L of the fluid channel [96]. For considering the effect of an entrance length, common correlations are [12,27,96]:

(41) $$\frac{Nu}{Nu_{\infty }}=1+6\frac{d_{h}}{L},$$

(42) $$\frac{Nu}{Nu_{\infty }}=1+{\left(\frac{d_{h}}{L}\right)}^{0.667},$$

(43) $$\frac{Nu}{Nu_{\infty }}=4/3\cdot {\left(\frac{d_{h}}{L}\right)}^{0.055},$$

where Nu is the enhanced Nusselt number in short fluid channels and $Nu_{\infty }$ is the Nusselt number in an infinitely long channel. However, Equations (41)–(43) are valid only for flows with a stationary (non-mixing) flow field [98]. In spacer-filled channels, the entrance region was estimated to be equal to half of the mesh size [99]. Furthermore, Li et al. [100] performed computational fluid dynamics (CFD) simulations and showed that an entrance length is equivalent to about three to five repeated flow cells in the mean flow direction. Thus, it was concluded that the entrance effects are negligible in spacer-filled channels [16,100]. 

In addition to the improved calculation of the heat transfer coefficients, better knowledge of the Nusselt number also enables a more precise determination of the concentration polarization. According to the film model [27,68,69,70,73,101,102,103,104], the feed concentration at the membrane surface $c_{fm}$ may be calculated as

(44) $$c_{fm}=(c_{f}-c_{p})\cdot \exp\left(\frac{\dot{m}}{\rho \beta }\right)\approx c_{f}\cdot \exp\left(\frac{\dot{m}}{\rho \beta }\right)$$

where $c_{f}$ is the feed bulk concentration, $c_{p}$ is the permeate bulk concentration, $\dot{m}$ is the mass flux and β the mass transfer coefficient. In MD modelling, the permeate bulk concentration is often considered negligible. The mass transfer coefficient β can be determined with the Sherwood number Sh, which is defined as

(45) $$Sh=\frac{\beta d_{h}}{D_{ij}}$$

where $D_{ij}$ is the binary diffusion coefficient. Profound knowledge of the Nusselt number simplifies the evaluation of the mass transfer coefficient according to the Chilton–Colburn analogy [105] between heat and mass transfer [43]:

(46) $$\frac{Sh}{{Sc}^{C_{3}}}=\frac{Nu}{{Pr}^{C_{3}}}$$

where Sc is the Schmidt number,

(47) $$Sc=\frac{\nu }{D_{ij}}.$$

## 3. Materials and Methods

In this study, heat transfer in spacer-filled channels was systematically investigated in a membrane distillation test rig for the concentration of seawater brines. The measurement data were evaluated and an empirical correlation for the Nusselt number was derived. Computational fluid dynamics simulations were performed to analyze the temperature differences within the MD channels. In the following, the MD test rig, the test procedure and the evaluation method of the measurement data are described.

### 3.1. Test Rig

The experiments were performed in a laboratory-scale MD test rig with a plate-and-frame module supplied by SolarSpring GmbH Membrane Solutions (Freiburg, Germany). The dimensions of the hot and cold fluid channels are 250 mm × 150 mm × 2 mm each. They are equipped with symmetrical 2 mm thick rhomboidal/diamond-type spacers (ϕ=70°, $l_{S}=5\;mm,$ $\varepsilon_{S}=80\%)$, providing an effective heat transfer area of 0.0375 m^2^. The hydraulic diameter of these spacer-filled channels is 1.83 mm, according to Equation (12).

**Figure 3:**
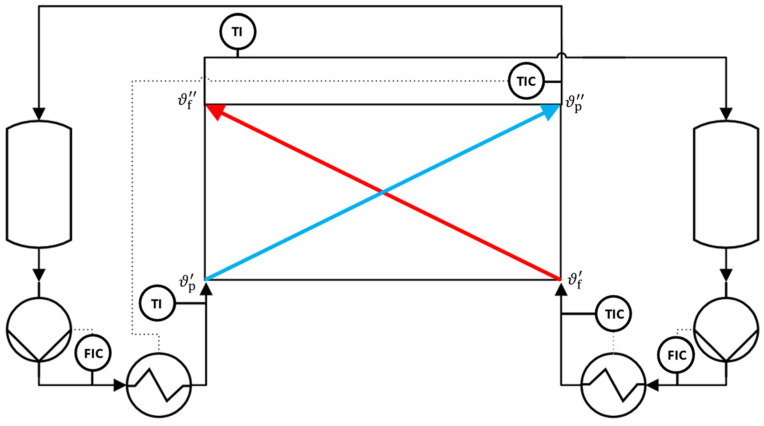
Schematic process diagram of the MD test rig configured for heat transfer measurements.

Figure 3 shows the schematic process diagram of the MD test rig configured for heat transfer measurements. The system is operated in a counter-current closed loop circulation driven by diaphragm pumps. The temperatures in the hot and cold fluid channels are separately controlled with external heat exchangers that are coupled to thermostats. In both fluid streams, inlet and outlet temperatures (Pt100; class A) as well as the inlet volume flow rates (OPTIFLUX 4040, uncertainty: ± 0.5% of reading, Krohne, Duisburg, Germany) are monitored. 

Instead of a membrane for MD experiments, an aluminum plate (Al 99.0/EN AW 1200) with a thickness of $\delta_{Al}=2\;mm$ was used to determine the heat transfer in spacer-filled channels. In contrast to a polymer membrane, the thermal conductivity of an aluminum plate (235 W/(m K) < $k_{Al}$ < 240 W/(m K)) can easily be measured and is well-known within a temperature range of 0 to 100 °C [31]. Moreover, the thermal resistance of the metal plate is low compared to the total heat transfer resistance and, thus, the convective heat transfer resistances predominate. In addition, the analysis of the heat transfer coefficients in the fluid channels is also facilitated by the omission of mass transfer phenomena. The module was thoroughly insulated.

In order to imitate seawater brines, sodium chloride (NaCl) solutions were used in both the feed and coolant channels to prevent the formation of scales on the heat transfer surfaces that would be expected to occur using real or artificial seawater brine samples [106]. The salinity in the feed channel was increased up to 95 g/kg, while the salinity in the permeate channel was kept at 1 g/kg. Sodium chloride of ACS grade was dissolved in deionized water. 

### 3.2. Test Procedure

The volume flow rate was varied between 50 and 300 L/h in steps of 25 L/h each, while maintaining the same reading in feed and coolant channels. In addition, the hot stream inlet temperature was varied from 30 to 80 °C, while the temperature difference between hot feed inlet and cold stream outlet was kept constant at 10 K. In order to achieve steady state, the system was thoroughly insulated. After a change in flow rate or temperature, the system was thermally equilibrated for at least one hour. Then, measurement data were recorded for 30 min with a sampling rate of 0.2 Hz. Mean values were used for evaluation.

### 3.3. Experimental Evaluation

In contrast to membrane distillation with a hydrophobic membrane, mass transfer from the feed side to the permeate side was prevented by using the aluminum plate during the heat transfer measurements. Thus, the evaluation follows the calculation of a heat exchanger. Energy balances applied to the feed and permeate streams result in

(48) $$\left|{\dot{Q}}_{f}\right|={\dot{M}}_{f}{\left.c_{p,f}\right|}_{{\bar{\vartheta }}_{f}}\left(\vartheta_{f}^{\prime }-\vartheta_{f}^{\prime \prime }\right)\hat{=}\left|{\dot{Q}}_{p}\right|={\dot{M}}_{p}{\left.c_{p,p}\right|}_{{\bar{\vartheta }}_{p}}\left(\vartheta_{p}^{\prime \prime }-\vartheta_{p}^{\prime }\right),$$

where $\dot{Q}$ is the heat flow rate and ${\left.c_{p}\right|}_{{\bar{\vartheta }}_{}}$ denotes the specific isobaric heat capacity at the stream’s mean temperature calculated from inlet $\vartheta^{\prime }$ and outlet $\vartheta^{\prime \prime }$ at the feed and permeate side, respectively. If the system is in steady state and considered as adiabatic, heat flow rates emitted by the feed and absorbed by the permeate should be equal. However, to counteract measurement inaccuracies, it is reasonable to determine a mean heat flow rate $\bar{\dot{Q}}$ for both fluid streams:

(49) $$\bar{\dot{Q}}=\frac{1}{2}\left(\left|{\dot{Q}}_{f}\right|+\left|{\dot{Q}}_{p}\right|\right).$$

In addition to balance equations, the heat flow rate in a counter-current setup is given as

(50) $$\dot{Q}=UA\;\frac{\left[\left(\vartheta_{f}^{\prime }-\vartheta_{p}^{\prime \prime }\right)-\left(\vartheta_{f}^{\prime \prime }-\vartheta_{p}^{\prime }\right)\right]}{\ln\left(\frac{\vartheta_{f}^{\prime }-\vartheta_{p}^{\prime \prime }}{\vartheta_{f}^{\prime \prime }-\vartheta_{p}^{\prime }}\right)},$$

where U is the overall heat transfer coefficient defined at surface A. The overall heat transfer resistance 1/(UA) is the sum of the single resistances due to convection in the feed boundary layer, conduction in the aluminum plate and convection in the permeate boundary layer [43,80], expressed as

(51) $$\frac{1}{UA}=\frac{1}{h_{f}\;A_{f}}+\frac{\delta_{Al}}{k_{Al}\;A_{m}}+\frac{1}{h_{p}\;A_{p}}$$

where $\delta_{Al}$ and $k_{Al}$ are thickness and thermal conductivity of the aluminum plate, respectively. It is valid to consider $A_{i}=\mathrm{const}$ for flat-sheet configuration in plate-and-frame modules. However, in tubular geometries $A_{f}$ and $A_{p}$ refer to the inner and outer surface, while $A_{m}$ is a log-mean surface area. During the measurements, the volume flow rates in the hot and cold channels were the same. Assuming that the influence of the temperature on the fluid properties is small and the heat transfer coefficients are similar in both fluid channels $\left(h_{f}=h_{p}\right)$, they may be represented by a mean heat transfer coefficient $\bar{h}$ [107], which can be expressed as

(52) $$\bar{h}=\frac{2}{\frac{1}{U}-\frac{\delta_{Al}}{k_{Al}}}\;.$$

This assumption seems reasonable for small temperature differences that just slightly alter the fluid properties in the fluid channels. A CFD case study was performed to prove that this assumption is justified in the present experimental configuration.

## 4. CFD Case Study

A CFD case study was carried out in order to examine the influence of temperature on the fluid properties and to prove the assumption of similar heat transfer coefficients in the hot and cold liquid streams when evaluating the measurement data. 

### 4.1. Setup

The 3D CFD model was implemented in ANSYS Fluent 19R3 and contains more than 10^6^ unit elements. Based on assumptions for steady-state, incompressible and laminar flow, the governing equations for the conservation of mass, momentum and energy are given as

(53) $$\nabla \cdot \vec{u}=0,\;\rho \left(\vec{u}\cdot \nabla \vec{u}\right)=-\nabla p+\nabla \cdot \overset{̿}{\tau }\;\mathrm{and}\;\nabla \left[\vec{u}\left(\rho E+p\right)\right]=\nabla \left(k\nabla \vartheta \right),$$

where the stress tensor $\overset{̿}{\tau }$ and total energy E are defined as

(54) $$\overset{̿}{\tau }=\left[\eta \left(\nabla \vec{u}+{\left(\nabla \vec{u}\right)}^{T}\right)\right]\;\mathrm{and}\;E=\int_{\vartheta_{0}}^{\vartheta }c_{p}dT+\frac{u^{2}}{2}\;.$$

Boundary conditions were set as follows: All walls adjacent to the fluid domains, including the heat transfer surface, were considered stationary walls with no-slip boundary condition. Except for the heat transfer surface, the fluid channel walls were assumed to be adiabatic, while the heat transfer surfaces facing the hot and cold fluid channels were thermally coupled to the adjacent fluid streams, allowing the conduction of heat from the hot to the cold side of the aluminum plate. The inlets were defined as mass flow inlets, which are equivalent to 50, 150 and 250 L/h (0.044, 0.132 and 0.220 m/s, respectively), while outlets were considered to be at ambient pressure. In order to achieve a constant driving temperature difference of 10 K at the hot stream inlet, the cold stream inlet temperature $\vartheta_{p}^{\prime }$ was iteratively adjusted until the temperature difference between hot stream inlet and cold stream outlet was 10 K. With respect to temperatures and fluid velocities, the fluid regime was considered as fully developed laminar. Temperature-dependent fluid properties were approximated by polynomial equations derived for seawater [24] in the temperature range of 0 to 100 °C and salinity range of 0 to 120 g/kg. The temperature-dependent thermal conductivity of the aluminum plate (Al 99.0/EN AW 1200) was implemented as a polynomial function derived from data given by Neubronner et al. [31]. Assuming that spacers lead to increased mixing and counteract the formation of thick boundary layers and, thus, the influence of temperature changes across the boundary layer on the fluid properties is more pronounced without spacers, they were not considered in the CFD study, because the purpose was to verify the assumption made in Equation (52).

### 4.2. Numerical Results and Discussion

The velocity and temperature distributions in the fluid channels were determined using ANSYS Fluent 19R3, as described in Section 4.1. The convective heat transfer coefficients and corresponding Nusselt numbers were calculated and analyzed.

**Figure 4:**
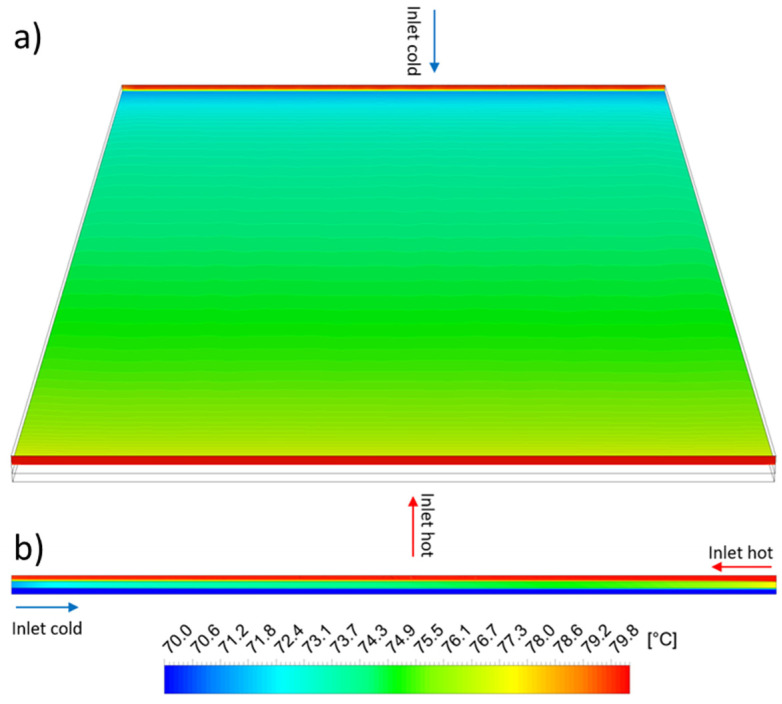
Temperature distribution (**a**) in the hot fluid domain at inlet, outlet and at the heat transfer surface; (**b**) in the cross-section in the center of hot fluid domain, aluminum plate and cold fluid domain. Volume flow rate $\dot{V}=50\;L/h$, hot fluid inlet temperature $\vartheta_{f}^{\prime }=80\;{}^{\circ}C$, cold fluid outlet temperature $\vartheta_{p}^{\prime \prime }=70\;{}^{\circ}C$.

Figure 4a shows the temperature distribution in the hot fluid domain. As given by the boundary condition, the temperature at the hot fluid inlet is evenly distributed. The temperature at the heat transfer surface is lowered by ongoing heat transfer and the formation of a fluid boundary layer. Furthermore, the heat transfer surface temperature decreases along the module length. As shown in Figure 4b, the temperature boundary layer develops from the inlet to the outlet in each stream.

**Figure 5:**
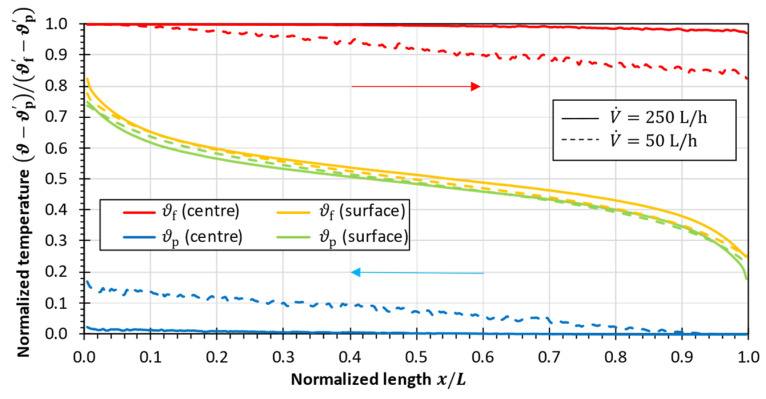
Normalized temperature profiles in the center of the fluid domain and at the heat transfer surface in the hot and cold fluid domains for ${\dot{V}}_{i}=50\;L/h$ ($\bar{Re}\approx 460$) and 250 L/h ($\bar{Re}\approx 2300$) with conduction through an aluminum plate. Channel length L=250 mm, hot stream inlet temperature $\vartheta_{f}^{\prime }=80\;{}^{\circ}C$, cold stream outlet temperature $\vartheta_{p}^{\prime \prime }=70\;{}^{\circ}C$. Arrows indicate fluid motion.

Figure 5 shows the calculation results for the temperatures in the channel center and at the heat transfer surface in the hot and in the cold fluid for a low and a high volume flow rate, respectively. In order to improve the comparability, the temperature profiles have been normalized with respect to the maximum temperature difference ($\vartheta_{f}^{\prime }-\vartheta_{p}^{\prime }$) which occurs in the module.

As shown in Figure 5, all the temperatures decline along the module length because of ongoing heat transfer. While the bulk temperatures in the center of the feed and permeate channels change insignificantly at the volume flow rate of 250 L/h, the bulk temperatures notably change at a low flow rate of 50 L/h. The temperatures at the heat transfer surface adjacent to the hot and cold fluid domains slightly differ, and the major temperature differences are established across the fluid boundary layers. Hence, the fluid boundary layers create the dominant resistances to the heat transfer.

**Figure 6:**
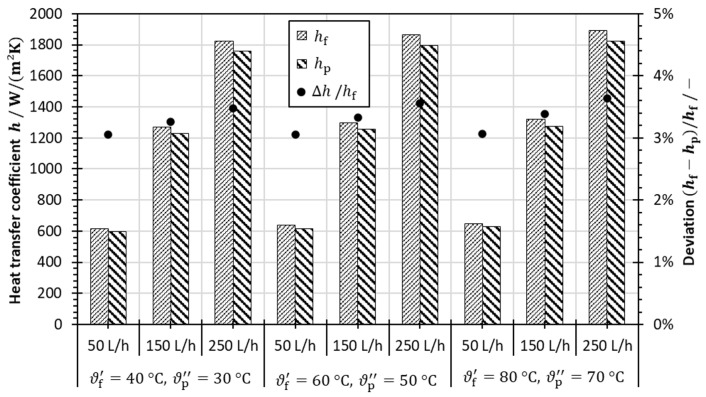
Heat transfer coefficients calculated using CFD for various volume flow rates ${\dot{V}}_{i}$ of 50, 150 and 250 L/h at different temperature levels and their deviations in the hot and cold fluid domains.

In order to analyze the effect of temperature level on the heat transfer in the hot and cold fluid domains, the heat transfer coefficients were evaluated. As shown in Figure 6, the surface heat transfer coefficients were calculated using ANSYS Fluent 19R3 for different operating temperature levels with hot stream inlet temperatures $\vartheta_{f}^{\prime }$ of 40, 60 and 80 °C, respectively. While a temperature difference of 10 K was maintained, the volume flow rate was adjusted to 50, 150 and 250 L/h (0.044, 0.132 and 0.220 m/s, respectively).

The heat transfer coefficients considerably increase with an increasing volume flow rate because forced convection is enhanced at higher fluid velocities. However, there is a less significant influence of the temperature level, as shown in Figure 6. For the same volume flow rates, the heat transfer coefficient is slightly higher at a higher temperature level and, thus, also in the hot stream fluid domain. This can be explained by a change in the temperature-dependent fluid properties affecting the Reynolds and Prandtl numbers. In general, higher temperatures lead to an increase in the Reynolds number and, hence, an increase in the heat transfer coefficient. However, the Prandtl number declines with higher temperatures and thus may reduce the heat transfer coefficient. Although these effects are counterbalancing each other, the results suggest that the slightly increased heat transfer coefficients are likely due to an elevated Reynolds number in the hot stream compared to the cold fluid domain. However, the temperature level affects the heat transfer only to a very small extent, as the deviation of the heat transfer coefficients between hot and cold fluid domain is calculated to be less than 4%.

**Figure 7:**
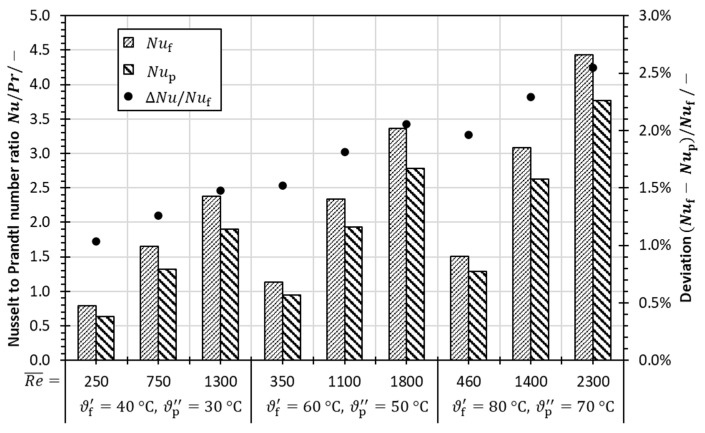
Nusselt-to-Prandtl-number ratios for various Reynolds numbers (averaged) and Nusselt number deviations in the hot and cold fluid domains based on CFD results.

Furthermore, the Nusselt number, which indicates the effectiveness of heat transfer, was evaluated based on the CFD results. Figure 7 shows the influence of the Reynolds number on the Nusselt number related to the Prandtl number for various temperature levels. An increase in the Nusselt-to-Prandtl-number ratio with increasing Reynolds number is obvious. Additionally, it is observed that the Nusselt-to-Prandtl-number ratio is reduced in the cold fluid domain. As the deviation of the Nusselt numbers, although increasing with Reynolds number, remains below 2.6%, the decreased Nusselt-to-Prandtl-number ratio in the cold fluid domain can be attributed to a higher Prandtl number at lower temperatures. Hence, it is reasonable to assume similar heat transfer coefficients and Nusselt numbers in the hot and cold fluid streams, which allows further evaluation of the heat transfer according to Equation (52) and confirms the procedure used by Sudoh et al. [107] when small temperature differences between hot and cold streams are applied.

## 5. Results and Discussion

The effects of both the variation of the fluid properties across the boundary layer and the fluid channel geometry $d_{h}/L$ on the Nusselt number were examined. In the following, the results are shown and discussed. Then, the heat transfer measurements are presented and discussed. Finally, a new empirical correlation for the Nusselt number is proposed that characterizes the heat transfer of seawater brines in spacer-filled channels.

### 5.1. Nusselt Number Correction for Variation of Fluid Properties and Channel Geometry

Due to the insignificant thermal resistance of the aluminum plate, the temperature differences across the fluid boundary layers are pronounced during heat transfer measurements. It was therefore investigated whether a correction of the Nusselt number has to be made to account for the change in the fluid properties. The thermophysical properties of seawater were calculated according to Nayar et al. [24].

**Figure 8:**
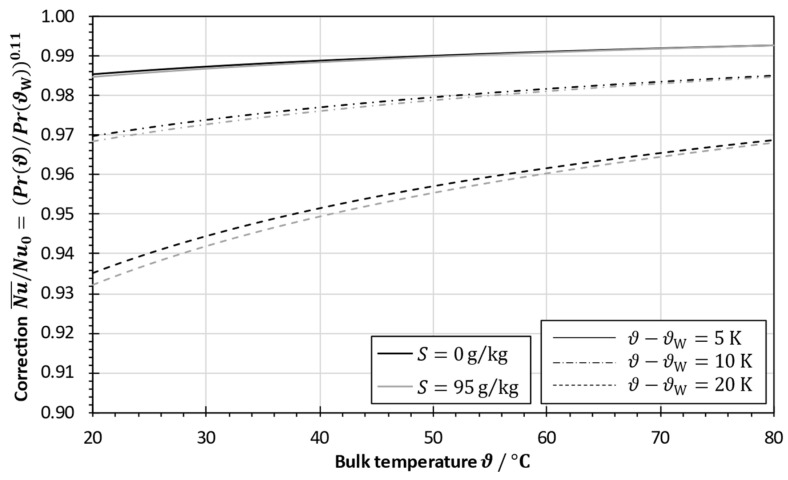
Influence of temperature level, temperature difference and salinity on Prandtl correction for empirical Nusselt correlations based on the fluid properties of seawater.

The correction of the Nusselt number was made according to Equation (40). Figure 8 shows the results as a function of the temperature level for different temperature differences and salinities. The correction becomes important at a lower temperature level and at a higher temperature difference between the fluid bulk ϑ and the heat transfer surface $\vartheta_{W}$. It is noteworthy that salinity has a small influence on the correction term. Since temperature differences bigger than 5 K across the fluid boundary layer at one side of the heat transfer surface are not reasonably applied in large-scale MD applications [19], it can be concluded that a correction for viscosity is not needed for heat transfer calculations at small temperature differences. However, if lab-scale systems are operated at very high temperature differences, a correction function must be considered.

**Figure 9:**
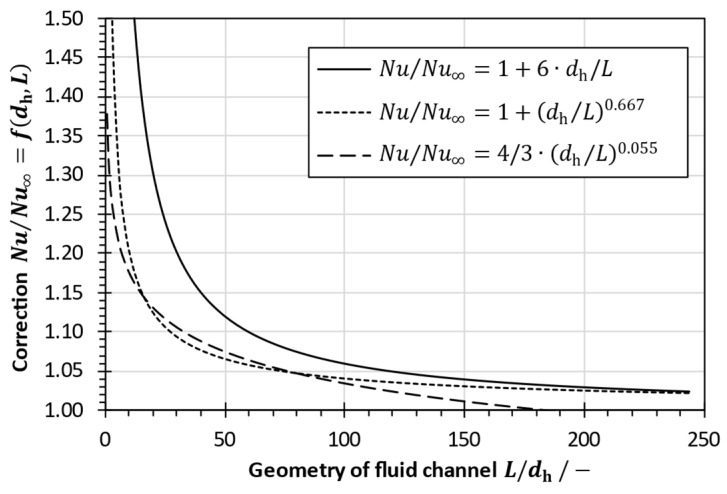
Influence of geometry of fluid channels on heat transfer enhancement.

Another principle that comes into effect is the heat transfer enhancement in the short entrance lengths of fluid channels. Heat transfer enhancement was determined using different correlations according to Equations (41)–(43). The results are shown in Figure 9. Heat transfer enhancement is significant for tubes that are short compared to their hydraulic diameter. However, the equations for the entrance length correction fade out for $L/d_{h}>50\;\mathrm{to}\;60$ [12,96]. In fact, enhancement of heat transfer is less than 5% at channel lengths $L/d_{h}>120$. 

Based on the elongated shape (typically $L/d_{h}>300\;\mathrm{to}\;5000$) of tubular MD modules, it can be concluded that entrance length effects can be neglected in heat transfer calculations in lumen geometries. Furthermore, the effects on the heat transfer in the entrance region of spacer-filled channels are assumed to be negligible [16,100].

Therefore, it is reasonable to simplify the Nusselt number correlation in Equation (13) by assuming $C_{4}=0$ for a given MD channel [16,33,78]. The Nusselt number for MD fluid channels can be expressed as

(55) $$Nu=C_{1}Re^{C_{2}}{Pr}^{C_{3}}.$$

The three unknown independent empirical constants $C_{1},C_{2}\;\mathrm{and}\;C_{3}$ can be obtained from a multi-dimensional regression of the experimental data. 

### 5.2. Evaluation of Heat Transfer Measurements and Derivation of a Correlation for Nusselt Number

Heat transfer coefficients can vary with fluid dynamics and properties. In the following, results are presented that were obtained in a plate-and-frame module with spacer-filled channels designed for membrane distillation, as described in Section 3.1. For determining the heat transfer coefficients, the inlet and outlet temperatures and the volume flow rates of both fluid streams were varied as described in Section 3.2. The evaluation of the heat transfer coefficient was conducted as explained in Section 3.3.

**Figure 10:**
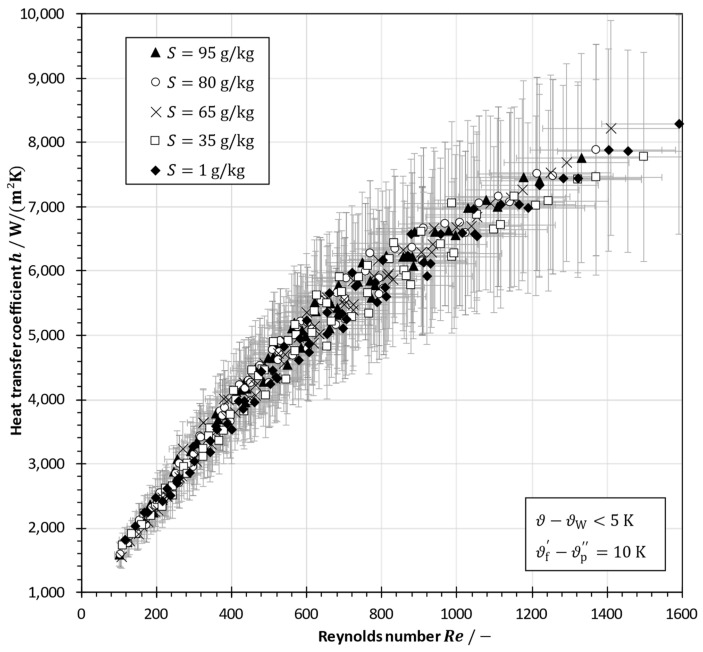
Heat transfer coefficients in a spacer-filled channel as a function of the Reynolds number with sodium chloride solutions having various salinities in a plate-and-frame module using an aluminum plate (error bars indicate the maximum error propagation estimation).

In Figure 10, heat transfer coefficients are depicted as a function of the Reynolds number for sodium chloride solutions with different salinities. As expected, they increase with Reynolds numbers from 1500 W/(m^2^K) at Re≈100 to 8300 W/(m^2^K) at Re≈1500. The error bars indicate the estimation of the maximum error propagation that results from measurement uncertainties. 

It is noticeable that the scattering of values tends to increase with rising Reynolds number. This scattering is a consequence of the changes in fluid properties due to different temperatures and salinities in various experiments leading to a variation of the Prandtl number, which was desired in order to develop a correlation with a broader range of application and validity. However, the influence of the flow rate, which determines the velocity and, thus, the Reynolds number, on the heat transfer coefficient is dominant. It should be noted that the fluid dynamics can be considered as fully developed turbulent except for Re<300.

**Figure 11:**
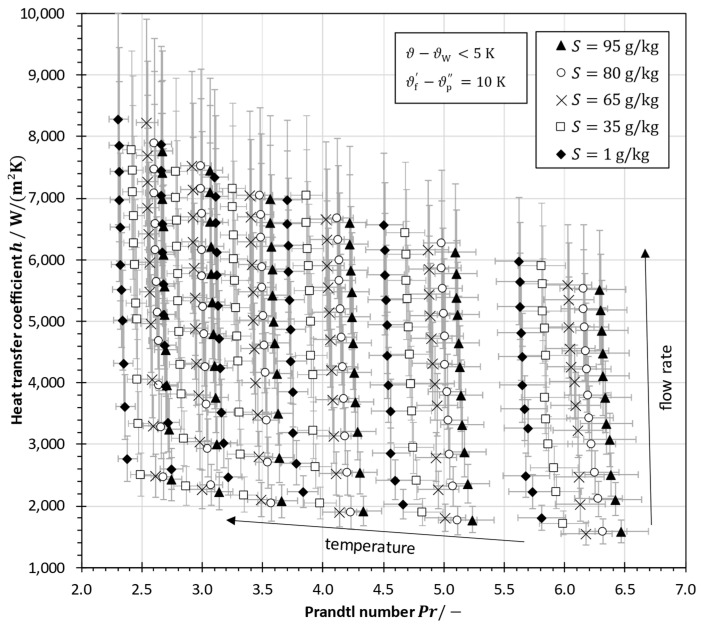
Heat transfer coefficients in a spacer-filled channel as a function of the Prandtl number with sodium chloride solutions having various salinities in a plate-and-frame module using an aluminum plate (error bars indicate the maximum error propagation estimation).

Figure 11 shows the influence of the variation of fluid properties in terms of the heat transfer coefficient as a function of the Prandtl number. As depicted in Figure 7, the heat transfer coefficient strongly depends on the flow rate that determines the Reynolds number. However, the effect of fluid properties is also important for properly analyzing the heat transfer. Clearly, the Prandtl number is a function of salinity but also of temperature. Although the increasing temperature leads to a decrease in the Prandtl number, for the same volume flow rate, the heat transfer coefficient is increased at higher temperatures due to an increase in the Reynolds number. In contrast to the Reynolds number, the Prandtl number is increased by an increase in salinity. However, the heat transfer coefficient is slightly reduced by an increase in salinity which shows that the Reynolds number plays a dominant role compared to the Prandtl number.

**Figure 12:**
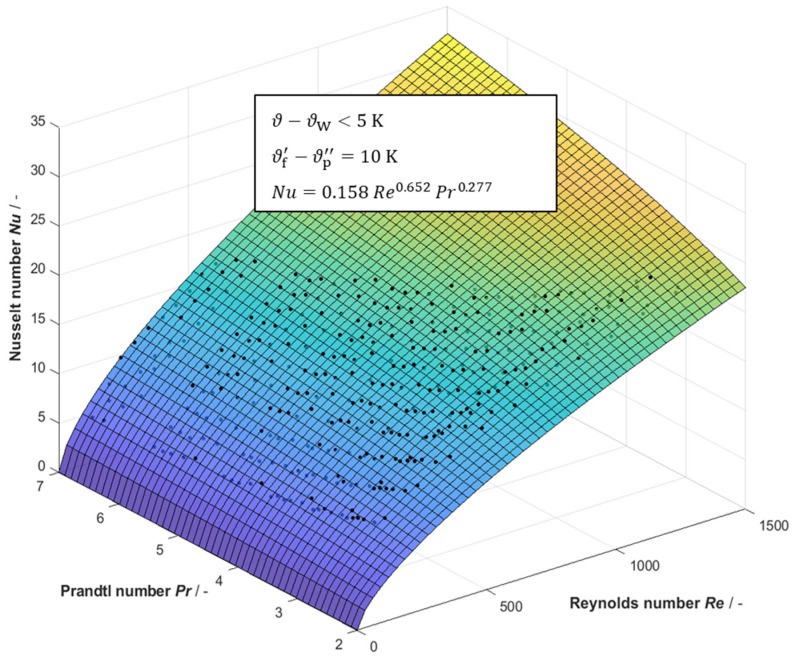
Evaluation of Nusselt numbers based on measured heat transfer coefficients with sodium chloride solutions (1–95 g/kg) simulating seawater brines and best fit based on least squares regression.

For the broad variation of Reynolds and Prandtl numbers, a multi-dimensional regression was carried out, in order to correlate the Nusselt number as a function of Reynolds number and Prandtl number. Least squares regression was performed in Matlab R2019 for the Nusselt numbers calculated from experimentally determined heat transfer coefficients for various Reynolds and Prandtl numbers. Figure 12 shows the results.

Applying a multi-dimensional regression based on Equation (55), it was found that the correlation

(56) $$Nu=0.158{Re}^{0.652}{Pr}^{0.277}$$

fits the experimental data best using a nonlinear least squares method in a Levenberg–Marquardt algorithm. The goodness of the parameter set for the fit function is characterized by the sum of squared errors SSE=44.474, the coefficient of determination $R^{2}=0.9936$ and the root-mean-square error RMSE=0.3688. Thus, Equation (56) represents a very good approximation. The 95% confidence bounds are given in Table 2. The given correlation is valid for 100<Re<1500 and 2<Pr<7 calculated with arithmetic mean temperatures between inlet and outlet of the feed and coolant channel, respectively.

**Figure 13:**
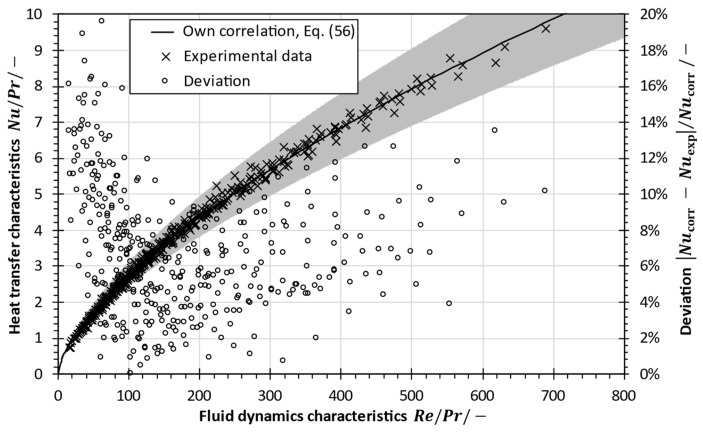
Comparison of the developed correlation in Equation (56) (shaded area indicates 95% confidence interval) with the authors’ experimental data and the deviation between the correlation and the experimental values.

The developed correlation in Equation (56) is compared to the experimental values in Figure 13. The ratio of Nusselt to Prandtl number indicates the heat transfer characteristics as a function of the fluid dynamics characteristics in terms of the Reynolds- to-Prandtl-number ratio. It is shown that the developed correlation approximates experimental data with good precision and the experimental data are well within the confidence bounds.

The absolute deviation between the experimental values and the developed correlation calculated at the same fluid characteristics indicates higher relative errors at lower Reynolds-to-Prandtl-number ratios (Re/Pr<100). This can be explained by changes in the flow regime at lower Reynolds numbers (180<Re<300), as described in Section 2.2. The deviation is lowest for Reynolds-to-Prandtl-number ratios around 200 and increases as a consequence of increasing experimental uncertainties, as also shown in Figure 10.

**Figure 14:**
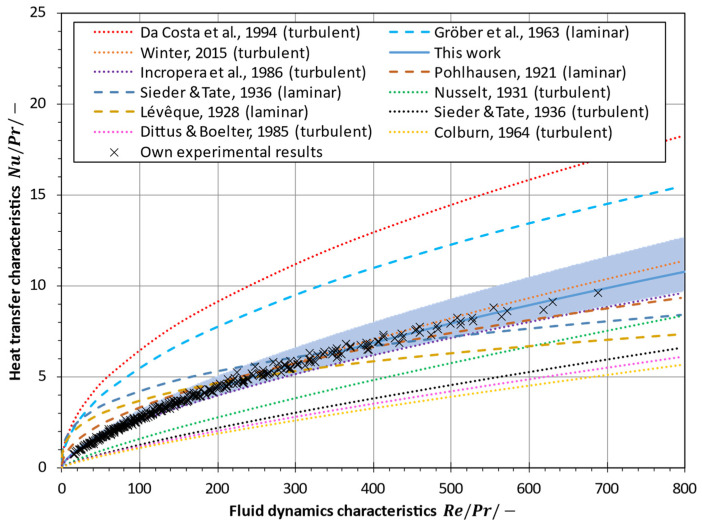
Heat transfer characteristics represented by the Nusselt-to-Prandtl-number ratio as a function of the fluid dynamics characteristics given by the Reynolds-to-Prandtl-number ratio for various heat transfer correlations used in MD modelling and for the newly developed correlation in Equation (56) compared to the authors’ experimental data (colored area is within the 95% confidence bounds) [16,40,57,58,75,77,81,85,87,88].

Figure 14 compares the newly derived Nusselt correlation in Equation (56) with various correlations for the Nusselt number that have been commonly used in MD modelling for flat-sheet configuration, as summarized in Section 2. The correlation found in this work yields relatively high heat transfer coefficients, which are only surpassed by correlations proposed by Da Costa et al. [40] and Gröber et al. [75]. The correlation presented in Equation (31) by Winter [16] shows very similar results, which can be explained by a comparable test setup and an equivalent spacer type inside the fluid channels. However, Winter [16] assumed $C_{3}=0.333$ for the exponent of the Prandtl number, while in this work $C_{3}=0.277$ was found experimentally. Nevertheless, Nusselt numbers calculated using Winter’s [16] correlation are well within the confidence interval.

Moreover, correlations developed for laminar flow overestimate the heat transfer coefficient at lower Reynolds numbers and underestimate it at higher Reynolds numbers. This could lead to a poor prediction of the heat transfer coefficient, especially at low Reynolds numbers (Re/Pr<100). 

Although flow is considered to be rather turbulent in spacer-filled channels, correlations for the Nusselt number that are commonly applied in the turbulent regime (Colburn [81], Dittus and Boelter [85], Nusselt [87] and Sieder and Tate [58]) severely understate the heat transfer coefficients found in this work. Hence, they may not be applicable in spacer-filled channels because of the prevailing lower Reynolds numbers. 

Furthermore, it is noteworthy that correlations used for laminar flow (Lévêque [57], Pohlhausen [77] and Sieder and Tate [58]) tend to yield higher Nusselt numbers compared to correlations commonly applied in the turbulent regime for the same Reynolds number. This underlines the shortcomings of empirical correlations derived for turbulent pipe flow when applied to spacer-filled channels. The results confirm that heat transfer characteristics need to be measured in spacer-filled channels and caution must be exercised when choosing an empirical correlation for the Nusselt number.

## 6. Conclusions

For modelling and simulation of membrane distillation processes that can be used for the concentration of seawater brines, a better knowledge of heat transfer in spacer-filled fluid channels is of the utmost importance. Therefore, a comprehensive literature review was performed on the heat transfer mechanisms in membrane distillation (MD) based on forced convection in the fluid streams and conduction through the membrane material. As the vapor is generated at the entrances of the pores and is condensed at the permeate side of the membrane, the mass transport also contributes to the heat transfer. The mass transport, in turn, is strongly dependent on the temperatures and temperature differences, leading to the coupling of heat and mass transfer.

The convective heat transfer is generally described by empirical correlations for the Nusselt number as a function of the Reynolds and Prandtl numbers. The empirical correlations have various forms and may also be linked to momentum transport. In this study, various empirical correlations for the Nusselt number were compared. It was found that the empirical correlations often used for MD simulation result in strongly varying Nusselt numbers that differ by up to an order of magnitude at low Reynolds numbers, compromising the accuracy of all further calculations.

Therefore, heat transfer was systematically studied in spacer-filled channels in a laboratory-scale MD test rig. Instead of a membrane, an aluminum plate was used for the heat transfer measurements. Both the feed and permeate channels were equipped with symmetrical 2 mm thick rhomboidal spacers. Numerous tests were carried out using sodium chloride solutions in a wide range of salinities between 1 g/kg and 95 g/kg and temperatures between 30 °C and 80 °C. The volume flow rates were varied between 50 L/h and 300 L/h. The experiments revealed high heat transfer coefficients in the range of 1500 to 8300 W/(m^2^K) for relatively low Reynolds numbers ranging from 100 to 1500, clearly indicating the influence of the spacers in the fluid channels on the heat transfer.

Computational fluid dynamics simulations were conducted to analyze the variations of fluid properties across the fluid boundary layer caused by a given temperature difference. It was found that the difference of heat transfer coefficients in the hot and cold fluid channels is below 4% when small driving temperature differences (ΔT=10 K) are applied. The results confirmed the procedure of evaluating the heat transfer measurements based on the assumption of similar heat transfer coefficients in the hot and cold fluid channels.

Then it was investigated whether the Nusselt number must be corrected to account for the variation of the fluid properties across the boundary layer and for the entrance length. Calculations showed that the influences of the varying fluid properties across the boundary layer and the entrance length are negligible for MD systems with lumen and flat-sheet geometries when being operated at small temperature differences (ΔT=10 K). 

The measurement data were evaluated and the empirical correlation

$$Nu=0.158{Re}^{0.652}{Pr}^{0.277}$$

was derived for 100<Re<1500 and 2<Pr<7. The empirical correlation represents the experimental data with a good accuracy, generally below 10% of deviation, which is slightly increased at lower fluid velocities.

In future work, the effects of the spacer type and geometry should be evaluated. In addition, it would be helpful to follow the approach of coupling heat and momentum transport in spacer-filled channels, which was successfully applied in plate heat exchangers [88]. In this study, a rigid aluminum plate between the hot and cold fluid channels was used for heat transfer measurements. The effect of a less stiff and rigid heat transfer material, similar to a membrane, should be studied in future work.

## Figures and Tables

**Table 1:** Empirical correlations for the friction factors in smooth circular tubes.

Correlation for the Friction Factor		Comment	Reference
$\zeta =0.316/{Re}^{0.25}$	(33)	Blasius equation $3\cdot 10^{3}\leq Re\leq 10^{5}$	[80,92,93]
$\zeta =0.184/Re^{0.2}$	(34)	Moody equation $10^{4}\leq Re<10^{6}$	[92,93]
$\zeta =0.0054+0.3964/Re^{0.3}$	(35)	Hermann equation $2\cdot 10^{4}\leq Re\leq 2\cdot 10^{6}$	[92]
$\zeta ={\left[1.8{log}_{10}\left(Re\right)-1.5\right]}^{-2}$	(36)	Konakov equation $10^{4}\leq Re\leq 10^{6}$	[37]
$\zeta ={\left[1.82\log_{10}\left(Re\right)-1.64\right]}^{-2}$ $={\left[0.79\ln\left(Re\right)-1.64\right]}^{-2}$	(37)	Filonienko equation $3\cdot 10^{3}\leq Re\leq 5\cdot 10^{6}$	[27,43,80,90,92,94]
$\zeta ={\left[1.2776\log_{10}\left(Re\right)-0.406\right]}^{-2.246}$	(38)	Taler equation $3\cdot 10^{3}\leq Re\leq 10^{7}$	[92]

**Table 2:** Empirical constants in the Nusselt number correlation in Equation (56) resulting from multi-dimensional regression based on Equation (55) with 95% confidence bounds.

Constant	Value	95% Lower Bound	95% Upper Bound
$C_{1}$	0.1580	0.1491	0.1669
$C_{2}$	0.6521	0.6450	0.6592
$C_{3}$	0.2767	0.2656	0.2877

## Data Availability

The data presented in this study are available on request by the corresponding author.

## Nomenclature

**Symbol**	**Description**	**Unit**
A	Area	$m^{2}$
C	Empirical constant	-
$C_{m}$	Membrane parameter	$kg/\left(m^{2}s\;Pa\right)$
c	Concentration	mol/L
$c_{p}$	Specific isobaric heat capacity	J/(kg K)
$D_{ij}$	Binary diffusion coefficient	$m^{2}/s$
d	Diameter	m
E	Specific total energy	J/kg
$f_{}$	Factor or function	div.
g	Universal gravitational constant	$m/s^{2}$
Gr	Grashof number	-
H	Height	m
h	Convective heat transfer coefficient	$W/(m^{2}K)$
$\Delta {\bar{h}}_{vap}$	Specific enthalpy of vaporization	J/kg
k	Thermal conductivity	W/(m K)
L	Length	m
$l_{S}$	Mesh size	m
$\dot{M}$	Mass flow rate	kg/s
$\dot{m}$	Mass flux	$kg/(m^{2}s)$
Nu	Nusselt number	-
P	Perimeter	m
p	Pressure	Pa
$p_{i}$	Partial pressure	Pa
Pr	Prandtl number	-
$\dot{Q}$	Heat flow rate	W
$\dot{q}$	Heat flux	$W/m^{2}$
$R^{2}$	Coefficient of determination	-
Ra	Rayleigh number	-
Re	Reynolds number	-
Ri	Richardson number	-
RMSE	Root-mean-square error	-
S	Salinity	g/kg
Sc	Schmidt number	-
Sh	Sherwood number	-
SSE	Sum of squared errors	-
U	Overall heat transfer coefficient	$W/(m^{2}K)$
u	Fluid velocity	m/s
$\vec{u}$	Fluid velocity vector	m/s
V	Volume	$m^{3}$
$\dot{V}$	Volume flow rate	$m^{3}/s$
x	Longitudinal dimension	m
β	Convective mass transfer coefficient	m/s
$\beta_{V}$	Coefficient of thermal expansion	1/K
δ	Thickness	m
ε	Porosity	-
ζ	Friction factor	-
η	Dynamic viscosity	Pa s
ϑ	Temperature	°C
ν	Kinematic viscosity	$m^{2}/s$
ρ	Density	$kg/m^{3}$
τ	Tortuosity	-
$\overset{̿}{\tau }$	Stress tensor	$N/m^{2}$
ϕ	Angle	°
**Sub- and Superscript**	**Description**	
0	Initial, boundary	
Al	Aluminum plate	
corr	Correlation	
crit	Critical	
exp	Experimental	
f	Feed bulk	
flux-law	Flux law	
forced	Forced convection	
fm	Feed–membrane interface	
free	Free convection	
g	Gaseous	
geometric mean	Geometric mean	
h	Hydraulic	
iso-strain	Iso-strain	
iso-stress	Iso-stress	
iso-superposed	Superposed iso-strain/iso-stress	
L	At length L	
lm	Logarithmic mean	
m	Membrane	
modified iso-strain	Modified iso-strain	
p	Permeate bulk	
pm	Permeate–membrane interface	
S	Spacer	
s	Solid	
W	Wall	
x	In direction of x	
${}^{\prime }$	Inlet	
${}^{\prime \prime }$	Outlet	
∞	Infinite length	
$\bar{}$	Average, mean	

## Appendix A

**Table A1:** Basic forms of empirical correlations for the Nusselt number, without modifications for viscosity changes or entrance lengths, used in MD modelling. Friction factor ζ may be calculated according to an appropriate correlation, as given in Table 1.

Nusselt Correlation		Application in MD Modelling	References
$\bar{Nu}=0.097\;Re^{0.73}{Pr}^{0.13}$	(A1)	Laminar, flat-sheet/tubular (lumen side)	[2,5,13,22,27,59]
$\bar{Nu}=0.152\;Re^{0.695}{Pr}^{0.333}$	(A2)	Flat-sheet, experimentally found for symmetric spacer (thickness 2.0 mm)	[16]
$\bar{Nu}=0.162\;Re^{0.656}{Pr}^{0.333}$	(A3)	Flat-sheet, experimentally found for symmetric spacer (thickness 3.2 mm)	[16]
$\bar{Nu}=0.15\;Re^{0.333}{Pr}^{0.43}Gr^{0.1}\;$	(A4)	Laminar, flat-sheet	[13]
$\bar{Nu}=0.298\;Re^{0.646}\;{Pr}^{0.316}$	(A5)	Laminar, flat-sheet/tubular (lumen side)	[13,59,108,109]
$\bar{Nu}=0.430\;Re^{0.591}\;{Pr}^{0.303}\;$	(A6)	Laminar, flat-sheet	[13]
$\bar{Nu}=0.664\;Re^{0.5}\;Pr^{0.333}$	(A7)	Laminar, 1<Pr<10, flat-sheet, spacer not inducing change of flow direction	[80,96,110]
$\bar{Nu}=0.664Re^{0.5}Pr^{0.333}{\left(d_{h}/L\right)}^{0.5}$	(A8)	Laminar, flat-sheet	[13,39]
$\bar{Nu}=0.644f_{S}{Re}^{0.5}{Pr}^{0.333}{\left(2d_{h}/l_{S}\right)}^{0.5}\;$	(A9)	Flat-sheet, spacer inducing change of flow direction, transition to fully developed turbulent $f_{S}=1.654{\left(d_{f}/\delta_{S}\right)}^{-0.039}\varepsilon_{S}^{0.75}{\left(\sin\left(\phi /2\right)\right)}^{0.086}$ $l_{S}$ mesh size; $d_{f}$ filament size; $\delta_{S}$ spacer thickness; $\varepsilon_{S}$ spacer voidage; ϕ angle between spacer filaments	[5,11,16,27,32,39]
$\bar{Nu}=0.74Re^{0.2}{\left(Gr\cdot Pr\right)}^{0.1}Pr^{0.2}$	(A10)	Laminar, flat-sheet	[13,108,109,111]
$\bar{Nu}=1.04\;{Re}_{}^{0.4}\;{Pr}_{}^{0.36}$	(A11)	Laminar, tubular (shell side cross-flow)	[62,63,112,113]
$\bar{Nu}=1.62\;{\left(Re\;Pr\;d_{h}/L\right)}^{0.333}$	(A12)	Laminar, 0.6<Pr<5, flat-sheet/tubular (lumen side)	[2,5,6,18,20,59,60,66,67,80,108,109,114]
$\bar{Nu}=1.62\left(1+0.015Gr^{0.333}\right){\left(Re\;Pr\;d_{h}/L\right)}^{0.333}$	(A13)	Laminar, flat-sheet	[13]
$\bar{Nu}=1.86\;{\left(Re\;Pr\;d_{h}/L\right)}^{0.333}$	(A14)	Laminar, $Re\;Pr\;d_{h}/L>10$, 0.6<Pr<5, flat-sheet/tubular	[3,6,13,27,57,61,62,63,65,68,70,71,72,73,74,80,108]
$\bar{Nu}=1.86{{Re}^{0.58}\left(Pr\cdot d_{h}/L\right)}^{0.333}$	(A15)	Laminar, experimentally found, flat-sheet	[17,108]
$\bar{Nu}=1.95\;{\left(Re\;\mathrm{Pr}\;d_{h}/L\right)}^{0.333}$	(A16)	Laminar, flat-sheet	[27]
$\bar{Nu}=C\;(R{e\cdot Pr)}^{0.23}{\left(d_{h}/L\right)}^{0.5}$	(A17)	Laminar, flat-sheet $C=\left\{\begin{array}{c} 15.0\;:\;\vartheta_{W}>\vartheta \;(heating)\\ 11.5\;:\;\vartheta_{W}<\vartheta \;(cooling) \end{array}\right.$	[13,27]
$\bar{Nu}=C+\frac{0.0668{Re\;Pr\;d}_{h}/L}{1+0.04{\left({Re\;Pr\;d}_{h}/L\right)}^{0.667}}$	(A18)	Laminar, Pr>5, tubular (lumen side) $C=\left\{\begin{array}{c} 4.36\;:\;{\dot{q}}_{W}=\mathrm{const}\\ 3.66\;:\;\vartheta_{W}=\mathrm{const} \end{array}\right.$	[2,5,6,12,18,43,80,115,116]
$\bar{Nu}=C+\frac{0.036{Re\;Pr\;d}_{h}/L}{1+0.0011{\left({Re\;Pr\;d}_{h}/L\right)}^{0.8}}$	(A19)	Laminar, tubular (lumen side) $C=\left\{\begin{array}{c} 4.36\;:\;{\dot{q}}_{W}=\mathrm{const}\\ 3.66\;:\;\vartheta_{W}=\mathrm{const} \end{array}\right.$	[3,6,18,27,84,108,111,114,117]
$\bar{Nu}=0.116\left(Re^{0.667}-125\right){Pr}^{0.333}$	(A20)	Transition, tubular (lumen side)	[2,5,18,59]
$\bar{Nu}=0.023\;Re^{0.8}\;{Pr}^{0.333}$	(A21)	Turbulent, 0.7<Pr<160, flat-sheet/tubular (lumen side)	[5,10,12,13,19,22,27,59,60,80,82,83,84,97,108,118,119,120]
$\bar{Nu}=0.023\;Re^{0.8}Pr^{n}$	(A22)	Turbulent, 0.7<Pr<160, flat-sheet/tubular (lumen side) $n=\left\{\begin{array}{c} 0.4\;:\;\vartheta_{W}>\vartheta \;(heating)\;\\ 0.3\;:\;\vartheta_{W}<\vartheta \;(cooling) \end{array}\right.$	[2,6,18,61,65,73,74,80,86,96,108]
$\bar{Nu}=0.023\;Re^{0.875}{Pr}^{0.25}\;$	(A23)	Turbulent, flat-sheet	[6,66,121]
$\bar{Nu}=0.027\;Re^{0.8}\;{Pr}^{0.333}$	(A24)	Turbulent, $0.7<Pr<1.67\cdot 10^{4}$, flat-sheet/tubular (lumen side)	[3,5,6,18,22,27,43,59,71,80]
$\bar{Nu}=0.027\;Re^{0.8}Pr^{n}$	(A25)	Turbulent, flat-sheet $n=\left\{\begin{array}{c} 0.4\;:\;\vartheta_{W}>\vartheta \;(heating)\;\\ 0.3\;:\;\vartheta_{W}<\vartheta \;(cooling) \end{array}\right.$	[22,108,122]
$\bar{Nu}=0.036\;Re^{0.8}{Pr}^{0.333}$	(A26)	Turbulent, flat-sheet	[22]
$\bar{Nu}=0.036Re^{0.8}{Pr}^{0.333}{\left(d_{h}/L\right)}^{0.055}$	(A27)	Turbulent, flat-sheet/tubular (lumen side)	[5,27,59,80,108]
$\bar{Nu}=0.036Re^{0.96}{Pr}^{0.333}{\left(d_{h}/L\right)}^{0.055}$	(A28)	Turbulent, flat-sheet, experimentally found	[17,108]
$\bar{Nu}=0.042\;{Re}^{0.59}\;{Pr}^{0.333}$	(A29)	Turbulent, tubular (shell side), experimentally found	[18]
$\bar{Nu}=0.13\;Re^{0.64}Pr^{0.38}$	(A30)	Turbulent, flat-sheet/tubular (lumen side)	[2,5,13,22,27,59,83,88,119]
$\bar{Nu}=0.206\;{\left(Re\cdot \cos\left(\phi \right)\right)}^{0.63}\;{Pr}^{0.36}$	(A31)	Turbulent, tubular (shell side, simultaneous cross- and parallel flow) 0°<ϕ<90°, $\phi =\left\{\begin{array}{c} 0{}^{\circ}\;:\mathrm{pure}\;\mathrm{cross}\text{-}\mathrm{flow}\\ 90{}^{\circ}\;:\mathrm{pure}\;\mathrm{parallel}\;\mathrm{flow} \end{array}\right.$	[2,18]
$\bar{Nu}=0.71\;{Re}_{}^{0.5}\;{Pr}_{}^{0.36}$	(A32)	Turbulent, tubular (shell side crossflow)	[62,63,112,113]
$\bar{Nu}=\frac{\left(\zeta /8\right)\left(Re-1000\right)Pr}{1+12.7\sqrt{\zeta /8}\left({Pr}^{0.667}-1\right)}$	(A33)	Turbulent, 0.5≤Pr≤2000, flat-sheet	[22,27,43,89,90]
$\bar{Nu}=\frac{\left(\zeta /8\right)Re\;Pr}{1.07+12.7\sqrt{\zeta f/8}\left({Pr}^{0.667}-1\right)}$	(A34)	Turbulent, 0.5<Pr<200, flat-sheet	[22,27,80]
$\bar{Nu}=\frac{\left(\zeta /8\right)Re\;Pr}{1.07+\frac{900}{Re}-\frac{0.63}{1+10Pr}+12.7\sqrt{\zeta /8}\left({Pr}^{0.667}-1\right)}$	(A35)	Turbulent, flat-sheet	[22,80,89]
$\bar{Nu}=\frac{\left(\zeta /8\right)Re\;Pr}{1.2+13.2\sqrt{\zeta /8}\left({Pr}^{0.667}-1\right)}$	(A36)	Turbulent, flat-sheet	[27,123]
